# Protocol to benchmark and evaluate the status of imbalance measure using correlation, data complexity, and ablation analyses

**DOI:** 10.1016/j.xpro.2026.104500

**Published:** 2026-04-27

**Authors:** Julie R. Pivin-Bachler, Egon L. van den Broek

**Affiliations:** 1 Department of Information and Computing Sciences, Utrecht University, 3584 CC Utrecht, the Netherlands

**Keywords:** Bioinformatics, Health Sciences, Computer sciences

## Abstract

Machine learning struggles with imbalanced data. Although several mitigation approaches exist, their application depends on the extent of imbalance. To determine the latter, a protocol was developed. Across 428 synthetic and 70 real datasets, 8 imbalance measures were benchmarked and evaluated using multiple classifiers, metrics, and correlation coefficients. The coding environment, data preparation, and correlation and complexity analyses are described. These are complemented by procedures for an ablation study of the most efficient measure: SIMBA (status of imbalance).

For complete details on the use and execution of this protocol, please refer to Pivin-Bachler et al.^1^

## Highlights

• Procedure for critical review and evaluation of imbalance measures
• Instructions for correlation analyses on synthetic and real datasets
• Steps for running data complexity and ablation analyses
• Guidance on how to use additional classifiers, evaluation metrics, and datasets

## Abstract (editor highlights)

Publisher’s note: Undertaking any experimental protocol requires adherence to local institutional guidelines for laboratory safety and ethics.

## Graphical abstract

**Figure:**
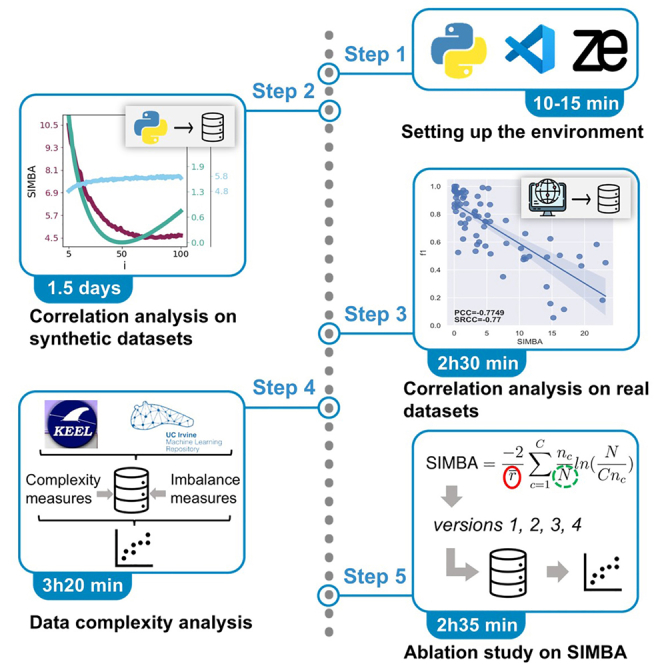

## Before you begin

Data is the fuel of machine learning. With imbalanced data, models struggle to learn patterns for the minority classes. Despite its prevalence in real-world data, imbalance is frequently overlooked or handled with an inadequate method. Indeed, methods to alleviate the impact of imbalance exist, but the choice of method to apply depends on the extent of imbalance. Seven imbalance measures were developed – namely, the imbalance ratio (IR), the adjusted IR (Adj-IR),^2^ the entropy of class proportions (C1),^3^^,^^4^ the multiclass imbalance ratio (C2),^4^^,^^5^ the imbalance degree (ID),^6^ the likelihood ratio imbalance degree (LRID),^7^ and the imbalance factor (IF)^8^ – but all present limitations. From this observation, the Status of IMBAlance (SIMBA) was created.^1^ Our protocol shows how to critically review these seven measures, evaluate their performance, and compare them to SIMBA.

Contrary to other existing imbalance measures, SIMBA considers data distribution and data overlap, both crucial elements to estimate the impact of imbalance on classification difficulty. More specifically, for data distribution, SIMBA includes a comparison between the actual distribution of a dataset and a perfectly balanced distribution, and incorporates the correlation between features and class labels to assess data overlap. SIMBA can be applied to datasets with any number of classes, features, and samples, making it a robust and generalizable measure.^1^

Imbalance measures are benchmarked via correlation analysis with classification performance; first, on synthetic data to study trends in controlled scenarios, then, on real data to assess performance under more complex conditions. Further, as imbalance measures represent only one aspect of data complexity,^4^ a data complexity analysis is performed to evaluate how well these measures indicate classification difficulty relative to other complexity measures. Finally, an ablation study on real data examines the importance of SIMBA’s three core components: namely, normalization, feature importance, and feature redundancy.^1^

In our protocol, all code was written in the Python programming language, and all tests were performed with the soft-/hardware described in the key resources table. Hence, the execution times concern experiments with this specific soft-/hardware and may vary depending on the employed hardware and operating system. All code was developed and tested on a Windows system. The workflow relies on the use of Visual Studio Code (VS Code), which is cross-platform, but has not been formally tested on Linux or macOS.

### Innovation

Imbalance measures should indicate classification difficulty,^9^ thus we analyzed their correlation with classification performance, with low correlation indicating poor capture of imbalance-related difficulty. Controlled synthetic scenarios highlight specific shortcomings across imbalance measures, revealing where they fail (e.g., unlike others, LRID lacks normalization and increases solely with dataset’s size). For the detailed shortcomings of imbalance measures, see our related manuscript.^1^ This motivated the development of SIMBA, built around 3 core components: normalization, feature information, and feature redundancy.

To examine the relationship between imbalance measures and data complexity, we include correlations with 20 data complexity measures.^2^ These reflect the degree of independence among them, while correlations with classification performance identify which measure best indicates classification difficulty. We also include an ablation study to further evaluate each component’s contribution in SIMBA’s formula. A component is deemed useful if its removal causes a noticeable drop in correlation (e.g., >0.1) between SIMBA score and classification performance.

For robustness, we include 5 classifiers, and Pearson (PCC) and Spearman (SRCC) correlation coefficients to consider strictly linear and monotonic relationships, respectively. For classification performance, while f1-score is one of the most common metrics for imbalanced datasets, we add the geometric mean (g-mean) to consider true negatives. Our evaluation includes 428 synthetic datasets with controlled variations on data and feature distribution. While most research proposing imbalance measure evaluate them on only 15 or 20 real datasets,^2^^,^^6^^,^^7^^,^^8^ we include 70 from distinct domains with 2 to 28 classes, 3 to 520 features, and 24 to 245,057 samples, ensuring generalizability.

### Set up the coding environment

**Timing:** 10–15 min

1. Install VS Code.
  a. Download VS Code for Windows (or your OS) from https://code.visualstudio.com/.
  b. Once downloaded, launch the.exe file, follow the instructions to install the software.
  c. Once installed, execute VS Code.

***Note:*** We recommend using the latest stable version available for VS Code. The protocol uses version 1.105.1.

**Figure 1:**
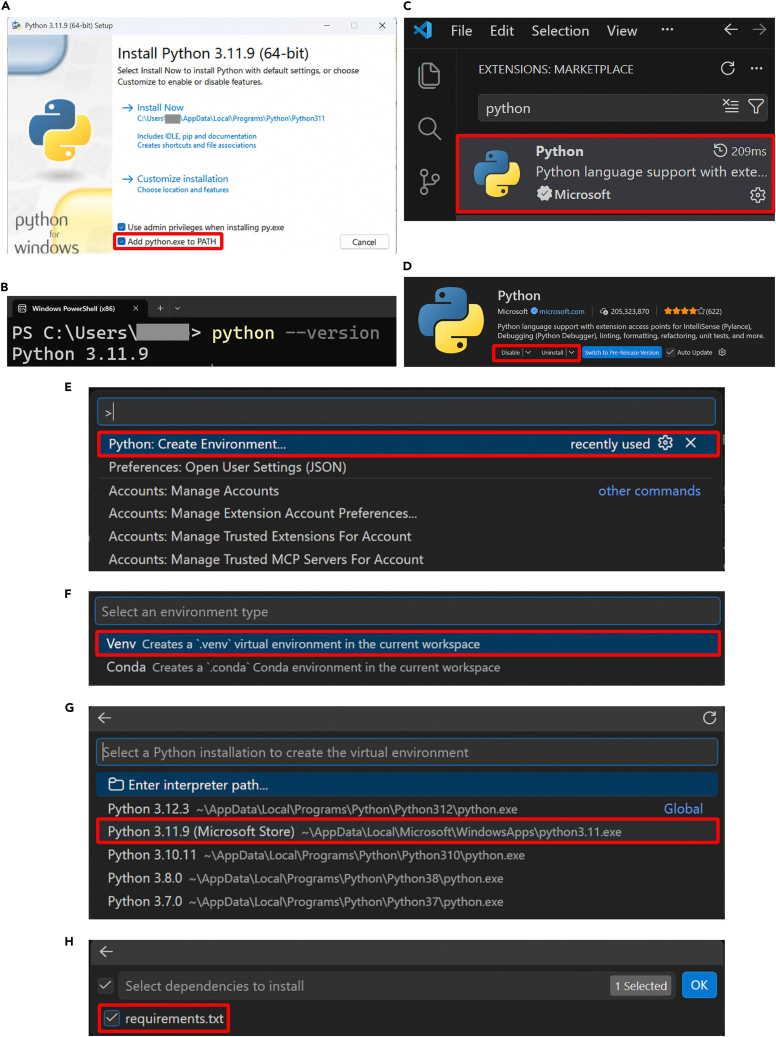
Step-by-step process for the set-up of the coding environment (A) Python installer dialog where the ‘Add to PATH’ option needs to be checked. (B) Verification of the Python installation on the Windows PowerShell terminal. (C) Python extension to install on Visual Studio Code. (D) Once installed, the python extension should appear installed and enabled leaving as available options ‘Disable’ and ‘Uninstall’ as shown within the red frame. (E) First step to create the virtual environment: select the ‘Python: Create Environment…’ command in the command palette. (F) Second step to create the virtual environment: select the Venv option. (G) Third step to create the virtual environment: select the Python version that you installed earlier. (H) Final step to create the virtual environment: select the *requirements.txt* file as dependencies to install.

***Note:*** Python is the only programming language used in this protocol. The protocol uses version 3.11.9. The Windows installer (64-bit) (or the installer for your OS) can be downloaded from https://www.python.org/downloads/release/python-3119.

**Figure 2:**
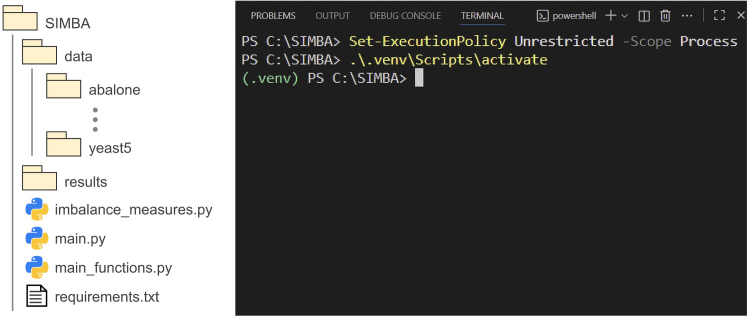
Required folder structure and virtual environment set up On the left, the folder structure required for the protocol to work is shown (with data and results subfolders, Python files, and the requirements file). On the right, the terminal screenshot displays how the terminal should look once the virtual environment is correctly activated.

2. Install Python. During installation, check the box “Add Python to PATH” before clicking “Install Now” (Figure 1A).

3. Verify Python has been correctly installed.
  a. Open a terminal or command prompt on your computer.
    ***Note:*** For Windows OS, you can do so by clicking the Windows icon and searching for ‘Windows PowerShell (x86)’.
  b. Type the following command:
    >python --version
    The result displayed should be Python 3.11.9 (Figure 1B). troubleshooting 1.
4. Create the virtual environment.
  a. Create a folder where you would like your project to be (e.g., *‘SIMBA’*).
    ***Note:*** In the rest of the protocol, we call this folder the SIMBA folder.
  b. Download the requirements.txt file from the Zenodo repository.^10^ Place the file in the SIMBA folder.
  c. Set up the virtual environment.
    i. Open the SIMBA folder in VS Code (click ‘File’ → ‘Open Folder’ and browse to your SIMBA folder).
    ii. Click the Extensions icon in the left side bar (or use [CTRL + Shift + X]).
    iii. Type ‘Python’ in the search bar, locate the extension published by Microsoft (Figure 1C).
    iv. Click Install (or Enable if it is already installed but disabled). When it is installed and enabled, it should appear as in Figure 1D.
    v. In VS Code, open the command palette with [CTRL+Shift+P] (or click ‘…’ → ‘Help’ → ‘Show All Commands’ on the tool bar at the top of the page).
    vi. Search and select the *‘Python: Create Environment’* command (Figure 1E).
    vii. Select.*Venv* (Figure 1F), and the interpreter *Python 3.11.9* that you have installed (Figure 1G).
    viii. Select *‘requirements.txt’* as dependencies to install (Figure 1H). troubleshooting 2.
      ***Note:*** If you cannot find the *‘Python: Create Environment’* command, you can open a terminal in VS Code (click View → Terminal) and type the command:
      >python -m venv .venv
      Afterwards, you can install the requirements as described in troubleshooting 2.
5. Activate the virtual environment.
  a. Open a terminal in VS Code by selecting View on the toolbar, then selecting Terminal.
  b. In the terminal, type the two following lines of command (press Enter after each line):
    >Set-ExecutionPolicy Unrestricted -Scope Process
    >.\.venv\Scripts\activate
    ***Note:*** This activates your virtual environment. If the environment is correctly set up and activated, you should see ‘(.venv)’ written on the top left corner of the terminal (see Figure 2), before the path of the SIMBA folder you are working in. Note that these lines of command are for a Windows OS. For a Linux or macOS, the first line is not required and the second line should be replaced by:
    >source .venv/bin/activate.

### Download the data and necessary code files

**Timing:** 1–2 min

6. Download the data.zip folder, and the Python files *main.py*, *main_functions.py*, and *imbalance_measures.py* from the Zenodo repository.^10^
7. Move the Python files and the data.zip folder into the SIMBA folder.
8. Extract the data.zip folder entirely into the SIMBA folder.
9. In the SIMBA folder, create a subfolder and name it ‘results’.

**CRITICAL:** The structure of the SIMBA folder must be as depicted in Figure 2.

## Key resources table

**Table:** 

REAGENT or RESOURCE	SOURCE	IDENTIFIER
**Deposited data**		

Processed 70 real datasets^10^	Zenodo	https://zenodo.org/doi/10.5281/zenodo.18792465
Datasets from UCI Machine Learning Repository^11^^,^^a^	UCI Machine Learning Repository	https://archive.ics.uci.edu/
Datasets from KEEL dataset repository^12^^,^^a^	KEEL repository	https://sci2s.ugr.es/keel/datasets.php

**Software and algorithms**		

Visual Studio Code	Microsoft	https://code.visualstudio.com/
Python 3.11.9	Python team	https://www.python.org/
Matplotlib 3.10.7	GitHub	https://github.com/matplotlib/matplotlib
Numpy 1.26.4	GitHub	https://github.com/numpy/numpy
Pandas 2.2.2	GitHub	https://github.com/pandas-dev/pandas
Pymfe 0.4.3	GitHub	https://github.com/ealcobaca/pymfe
Scikit-learn 1.5.1	GitHub	https://github.com/scikit-learn/scikit-learn
Scipy 1.14.0	GitHub	https://github.com/scipy/scipy
Seaborn 0.13.2	GitHub	https://github.com/mwaskom/seaborn
Python functions from Zenodo repository^10^: *main.py*, *main_functions.py*, and *imbalance_measures.py*	Zenodo	https://zenodo.org/doi/10.5281/zenodo.18792465

**Other**		

13th Gen Intel(R) Core(TM) i7-1360P (2.20 GHz CPU)	N/A	N/A
16GB RAM	N/A	N/A
Windows 11 (version 25H2) operating system	N/A	N/A

a If you use the pre-processed datasets from the Zenodo repository, this is not needed.

## Step-by-step method details

***Note:*** In the following, you will work solely with the *main.py* file (and *main_functions.py* file for optional steps), which you can open in VS Code.

***Note:*** The script is interactive. Some results are displayed sequentially in the terminal by imbalance measure, requiring the user to press ‘Enter’ to proceed to the next measure. Similarly, when multiple figures are generated, the user must press ‘q’ to close the current figure and proceed to the next one.

***Note:*** The protocol describes the steps necessary to reproduce the results from Pivin-Bachler et al.,^1^ but also provides optional steps for researchers who may want to test slightly different elements (e.g., modifying the synthetic datasets, adding/removing real datasets, and selecting a different classifier or classification evaluation metric). As each main step takes from 2 h 30 to 1.5 days, an optional fast-run minimal example is provided at the end of each main step, denoted by ‘[Fast-run minimal example]’, with its shorter duration. While only the full-scale experiments allow to reproduce the results from Pivin-Bachler et al.,^1^ these minimal examples can be used as a demonstration, or to verify the code is properly working before launching the full-scale longer steps. For strict reproduction of published results, run the full-scale experiments and ignore the optional steps.

### Correlation analysis on synthetic datasets

**Timing:** 1.5 days

**Timing:** 30 min (for step 1.b.i)

**Timing:** 25 min (for step 1.b.ii)

**Timing:** 1 day 11 h (for step 1.b.iii)

**Timing:** 1 h 15 min (for step 2.b.i)

**Timing:** 27 min (for step 2.b.ii)

**Timing:** 50 min (for step 2.b.iii)

**Timing:** 25 min (for optional fast-run minimal example)

Here, we describe the steps to benchmark the imbalance measures (IR, Adj-IR, C1, C2, ID, LRID, IF, and SIMBA) on synthetic datasets for varying data distribution and feature components. This is used to compute the correlation between imbalance measures and classification performance on controlled scenarios (steps 1 and 2) and visualize the imbalance measure trends (steps 3 and 4).

***Note:*** The synthetic data analysis includes two types of variations – data distribution and feature components – each with three scenarios. For data distribution, class sizes are altered in the scenarios to check the effects on the imbalance measure trends, while for feature components the focus is on the number of informative, non-informative, and redundant features (for a full description of the scenarios, we refer to Pivin-Bachler et al.^1^).

***Note:*** The parameters selected by default allow for reproducing the results from Pivin-Bachler et al.^1^ For the synthetic datasets, Support Vector Machines (SVM) are used with 5-fold cross-validation. As the generation of synthetic datasets involves randomness, each scenario is run 100×. Hence, the correlation results appear in the terminal with a mean and standard deviation over the 100 runs. Each correlation is calculated between the imbalance measures scores and the average classification performance on the 5 folds, either with the f1-score or g-mean.

1. First, we consider variations in data distribution. For this, in the main.py file, on line 23, set the action to:
  >action = ‘synth_data’
  a. To select the classification performance metric (between f1-score and g-mean), set the eval variable on line 24.
    >eval = ‘f1’
    or
    >eval = ‘gmean’
  b. Set the number of times each scenario should be run on line 25:
    >N = 100
    ***Note:*** By default, all scenarios are run 100× (N=100), to reproduce the study in Pivin-Bachler et al.^1^
    ***Note:*** By default, all 3 scenarios described in Pivin-Bachler et al.^1^ are run. To run scenarios separately, see the substeps i, ii, and iii below.
    i. To run only scenario a, set the variable scenarios on line 26:
      >scenarios = ‘a’
    ii. To run only scenario b, set the variable scenarios on line 26:
      >scenario
      s = ‘b’
    iii. To run only scenario c, set the variable scenarios on line 26:
      >scenarios = ‘c’
  c. To launch the program, in the terminal, type: troubleshooting 3.
    >python ./main.py
    ***Note:*** During execution, the current trial being run will be displayed in the terminal. Once all trials are completed, the results of the correlation analysis are displayed in the terminal. PCC and SRCC results appear one at a time per imbalance measure. To go to the next imbalance measure, press Enter in the terminal (if you prefer having all results at once, you can comment the ‘input()’ on line 1104 of the *main_functions.py* file).
2. The second type of variation concerns varying feature components. For this, in the main.py file, on line 23, set the action to:
  >action = ‘synth_ft’
  a. Select the classification performance metric as for step 1.a.
  b. Set the number of times each scenario should be run as for step 1.b.
    ***Note:*** As in the previous step, by default, all 3 scenarios described in Pivin-Bachler et al.^1^ are run. To run scenarios separately, see the substeps i, ii, and iii below.
    i. To run only scenario a, set the variable scenarios on line 26:
      >scenarios = ‘a’
    ii. To run only scenario b, set the variable scenarios on line 26:
      >scenarios = ‘b’
    iii. To run only scenario a, set the variable scenarios on line 26:
      >scenarios = ‘c’
  c. To launch the program, in the terminal, type: troubleshooting 3.
    >python ./main.py
    ***Note:*** During execution, the current trial being run will be displayed in the terminal. Once all trials are completed, the results of the correlation analysis are displayed in the terminal. PCC and SRCC results appear one at a time per imbalance measure. To go to the next imbalance measure, press Enter in the terminal (if you prefer having all results at once, you can comment the ‘input()’ on line 1104 of the *main_functions.py* file).
    ***Optional:*** If you wish to create a different scenario, whether on varying data distribution or on feature components, you can do so by modifying the parameters when calling the function run_synthdata (after line 55 in *main.py*). We recommend looking at the different parameters of the function run_synthdata in the file *main_functions.py* (line 244). troubleshooting 4.
3. Visualize trends on scenarios with varying data distribution. For this, in the main.py file, on line 23, set the action to:
  >action = ‘synth_data_plot’
  To launch the program, in the terminal, type: troubleshooting 3.
  >python ./main.py

4. Visualize trends on scenarios with varying feature components. For this, in the main.py file, on line 23, set the action to:
  >action = ‘synth_ft_plot’
  To launch the program, in the terminal, type: troubleshooting 3.
  >python ./main.py
  **CRITICAL:** Steps 3 and 4 only work if you have completed the correlation analysis on all scenarios with synthetic datasets. Having run all scenarios (a, b, and c) with varying data distribution allows step 3 to work, and all scenarios (a, b, and c) with varying feature components allows step 4 to work.
  ***Optional:*** Fast-run minimal example.
  Follow steps 1 to 4 as for the full-scale example but set the number of repetitions to 1 in steps 1 and 2, before launching the command.
  >python ./main.py
  in the terminal. To do so, in the *main.py* file, line 25, set:
  >N = 1

### Correlation analysis on real datasets

**Timing:** 2 h 30 min

**Timing:** 3 min (for optional fast-run minimal example)

This step is used to benchmark the imbalance measures on the real datasets contained in the data folder. The correlation between imbalance measures and classification performance is computed for different classifiers and visualized.

***Note:*** The correlation analysis is run for all imbalance measures (IR, Adj-IR, C1, C2, ID, LRID, IF, and SIMBA) with 5 different classifiers (SVM; Linear Discriminant Analysis – LDA; Random Forests – RF; k-Nearest Neighbours – kNN; and Multi-Layer Perceptron – MLP).

5. In the main.py file, on line 23, set the action to:
  >action = ‘real_data’
  a. Select the classification performance metric as for step 1.a.
    ***Optional:*** By default, the correlation analysis is performed with all 5 classifiers to reproduce the results of Pivin-Bachler et al.^1^ If you would like to remove some of the classifiers, you can do so on line 101. By default, you have:
    >clfs = [‘SVM’, ‘LDA’, ‘RF’, ‘kNN’, ‘MLP’]
    If, for instance, you would like to keep only SVM and RF, replace this with: troubleshooting 5.
    >clfs = [‘SVM’, ‘RF’]
    ***Note:*** The timing per classifier is as follows, for SVM: 1 h 42 min; for LDA: 3 min; for RF: 5 min; for kNN: 4 min; and for MLP: 36 min.
  b. To launch the program, in the terminal, type: troubleshooting 3.
    >python ./main.py
    ***Note:*** The correlation results are presented per classifier and should be examined as such to avoid potential classifier-specific biases to impact overall results and assess consistency. As each classifier has distinct learning principles, the correlation results vary per classifier, but the overall trend remains the same.^1^
    ***Optional:*** If you want to add another type of classifier that is not among the 5 implemented ones, you can do so with the *main_functions.py* file. More specifically, you can add an ‘elif’ statement in the get_eval_score function (see lines 96 to 110), and change the list of classifiers accordingly in the *main.py* file (line 101).
    ***Optional:*** If you want to add other real datasets, format them like the datasets present in the data folder. Create a new subfolder in the data folder and give it the dataset’s name. Add the name to the list of datasets in the *main_functions.py* file. For a binary dataset, add it to the list named ‘l_datasets_bin’ (line 738); for a multiclass dataset, add it to the list named ‘l_datasets’ (line 731). troubleshooting 6.
6. In the main.py file, on line 23, set the action to:
  >action = ‘real_data_plot’
  a. Select the classification performance metric as for step 1.a.
    **CRITICAL:** This step only works if you have completed the correlation analysis before on the real datasets. By default, it shows the results for each of the 5 possible classifiers. If you have run the previous step with the default parameters, make sure to use the same classification performance metric. If you have changed the list of classifiers in the previous step, do the same in this step by modifying line 110. troubleshooting 5.
  b. To launch the program, in the terminal, type: troubleshooting 3.
    >python ./main.py
    ***Note:*** The results appear in two ways. First, quantitative results in the terminal with PCC and SRCC for all, only binary, and only multiclass datasets for each classifier. Second, the regression lines in figures with binary and multiclass datasets separated, and all datasets together.
  c. Press Enter in the terminal to open the Figure.
  d. Press the key ‘q’ on the keyboard to close the figure and continue to the next classifier.
  e. Repeat sub-steps c and d until the results of all classifiers have been shown.
    ***Optional:*** Fast-run minimal example.
    Follow steps 5 and 6 as for the full-scale example but reduce the list of classifiers to LDA in step 5 before launching the command.
    >python ./main.py
    in the terminal. To do so, in the *main.py* file, line 101, set:
    >clfs = [‘LDA’]
    Make sure to do the same in step 6 by changing the list of classifiers to LDA in the *main.py* file, line 110.

### Data complexity analysis

**Timing:** 3 h 20 min

**Timing:** 15 s (for optional fast-run minimal example)

Here, we describe the steps to run the data complexity analysis to verify the relationship between all imbalance measures and data complexity measures.

**Figure 3:**
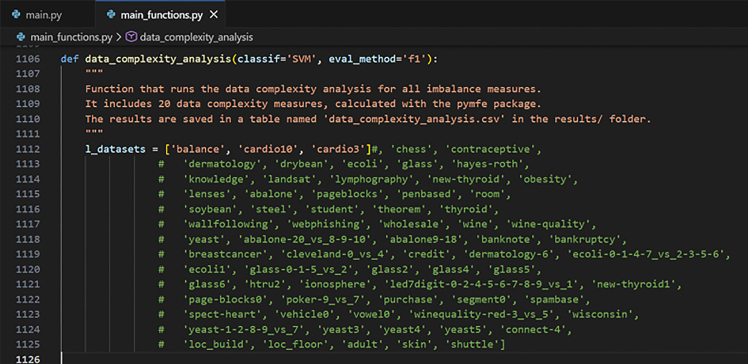
List of datasets in the *main_functions.py* file to run the minimal example of the data complexity analysis step (step 7)

7. In the main.py file, on line 23, set the action to:
  >action = ‘complexity’
  a. Select the classification performance metric as for step 1.a.
  b. Select the classifier (between SVM, LDA, RF, kNN, and MLP) by setting the ‘clf’ variable on line 123.
  c. To launch the program, in the terminal, type: troubleshooting 3.
    >python ./main.py
    **CRITICAL:** Make sure that lines 124 and 125 have the same values for the parameters ‘classif’ and ‘eval_method’. troubleshooting 7.
    ***Note:*** The results appear per data complexity measure. For each one, the correlation and associated p-value with the classification performance and with each imbalance measure are displayed. The imbalance measures appear in the following order: IR, Adj-IR, C1, C2, ID, LRID, IF, and lastly, SIMBA.
    ***Optional:*** If you want to use another type of classifier that is not among the 5 implemented ones, you can do so with the *main_functions.py* file. More specifically, you can add an ‘elif’ statement in the get_eval_score function (see lines 96 to 110), and change the classifier to use accordingly on line 123 of the *main.py* file.
    ***Optional:*** By default, the data complexity study is run on the 70 real datasets contained in the data folder. If you want to add other real datasets, format them like the datasets present in the data folder. Create a new subfolder in the data folder and give it the dataset’s name. Add its name to the list of datasets ‘l_datasets’ on line 1112 of the *main_functions.py* file. troubleshooting 5.
    ***Note:*** As described in Pivin-Bachler et al.,^1^ for large datasets (i.e., more than 30,000 samples or more than 20,000 samples combined with more than 100 features), the resources needed to run the ‘Data complexity analysis’ step were too high. Thus, for 6 out of the 70 real datasets (namely, 'connect-4′, 'loc_build', 'loc_floor', 'adult', 'skin', and 'shuttle'), only 10% of the datasets were kept to run the step. If you possess sufficient resources to run these datasets entirely, or would like to use a different percentage, you can change it in the *main_functions.py* file, see lines 1145 to 1149. The subsampling may slightly affect the numerical values of data complexity measures and hence, the resulting correlation estimates. However, given the large original sample sizes and the fact that subsampling was applied to only 6 out of 70 datasets, the impact of a different subsampling method on the overall correlation results is expected to be minor.
    ***Optional:*** Fast-run minimal example.
    Follow step 7 as for the full-scale example but keep only the first 3 datasets before launching the command.
    >python ./main.py
    in the terminal. To do so, in the *main_functions.py* file, comment all datasets listed in the variable ‘l_datasets’ on line 1112, except the 3 first ones (see Figure 3).

### Performing the ablation study

**Timing:** 2 h 35 min

**Timing:** 5 min (for optional fast-run minimal example)

Here, the steps described correspond to the ablation study on the SIMBA formula. Four versions of SIMBA (SIMBA whole and SIMBA without one of the three core components) are compared via correlation analysis on the real datasets.

***Note:*** The Timing mentioned corresponds to using eval = ‘f1’ and classif=‘SVM’. These default values also reproduce the results from Pivin-Bachler et al.^1^

8. In the main.py file, on line 23, set the action to:
  >action = ‘ablation’
  a. Select the classification performanc7e metric as for step 1.a.
  b. Select the classifier (between SVM, LDA, RF, kNN, and MLP) by setting the ‘classif’ parameter on line 116.
  c. To launch the program, in the terminal, type: troubleshooting 3.
    >python ./main.py
9. Once step 8 is completed, in the main.py file, on line 23, set the action to:
  >action = ‘ablation_plot’
  **CRITICAL:** You must leave the same classifier (line 119) and evaluation method (line 24) as in step 8. troubleshooting 8.
  To launch the program, in the terminal, type: troubleshooting 3.
  >python ./main.py
  ***Optional:*** If you want to use another type of classifier that is not among the 5 implemented ones, you can do so with the *main_functions.py* file. More specifically, you can add an ‘elif’ statement in the get_eval_score function (see lines 96 to 110) and change the classifier to use accordingly in the *main.py* file (lines 116 and 119).
  ***Optional:*** By default, the ablation study is run on the 70 real datasets contained in the data folder. If you want to add other real datasets, format them like the datasets present in the data folder. Create a new subfolder in the data folder and give it the dataset’s name. Add the name to the list of datasets in the *main_functions.py* file. For a binary dataset, add it to the list named ‘l_datasets_bin’ (line 849); for a multiclass dataset, add it to the list named ‘l_datasets’ (line 842). troubleshooting 5.
  ***Optional:*** Fast-run minimal example.
  Follow steps 8 and 9 as for the full-scale example but use LDA instead of SVM in step 8 before launching the command.
  >python ./main.py
  in the terminal. To do so, in the *main.py* file, line 116, set:
  >run_real_ablation(classif = ‘LDA’, eval_method=eval)
  Make sure to do the same in step 9 by using LDA instead of SVM in the *main.py* file, line 119:
  >plot_real_ablation(classif = ‘LDA’, eval_method=eval)

## Expected outcomes

**Figure 4:**
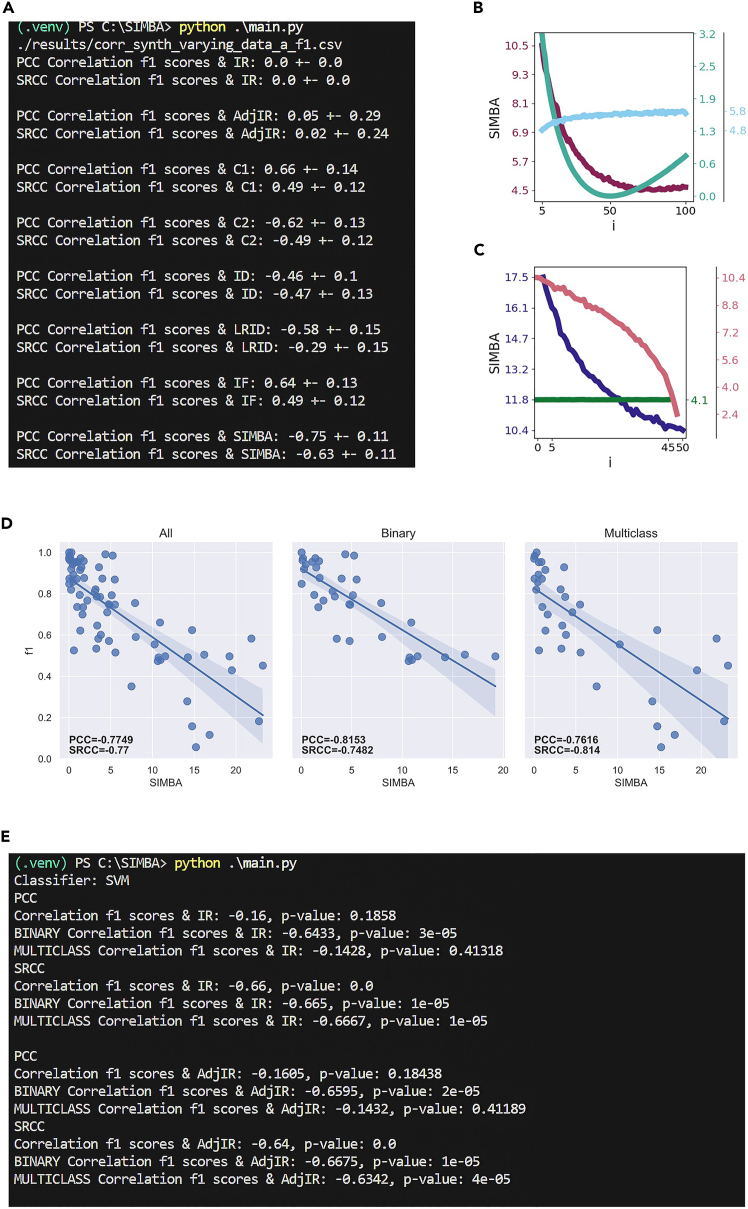
Expected outcomes in the terminal and in visual plots (A) Correlation results appearing in the terminal from scenario ‘a’, obtained with step 1.b.i. (B) Resulting plot for the SIMBA trends for synthetic datasets with varying data distribution, obtained with step 3. Figure adapted from Pivin-Bachler et al.^1^ (C) Resulting plot for the SIMBA trends for synthetic datasets with varying feature components, obtained with step 4. Figure adapted from Pivin-Bachler et al.^1^ (D) Resulting plot of step 6 for regression lines between SIMBA and f1-scores on all, only binary, and only multiclass real datasets. Pearson and Spearman correlation coefficients (PCC and SRCC) appear on each plot. Figure adopted from Pivin-Bachler et al.^1^ (E) Resulting Pearson and Spearman correlation coefficients (PCC and SRCC) appearing in the terminal for the first two imbalance measures (imbalance ratio and adjusted imbalance ratio, noted IR and Adj-IR) with the support vector machine (SVM) classifier, obtained with step 6.

**Figure 5:**
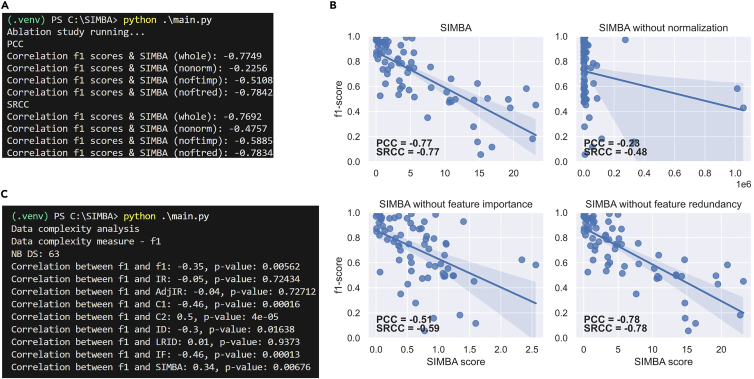
Expected outcomes in the terminal and in visual plots for the data complexity analysis and the ablation study (A) Pearson (PCC) and Spearman (SRCC) correlation coefficients appearing in the terminal for the ablation study, obtained with step 8. (B) Resulting plot for the ablation study on SIMBA, displaying its 4 versions: as a whole, without normalization, without feature importance, and without feature redundancy. This plot is obtained with step 9. Figure adopted from Pivin-Bachler et al.^1^ (C) Pearson correlation coefficients results and associated p-values for the first data complexity measure named ‘f1’. The number of considered datasets is displayed after ‘NB DS’ (datasets yielding NaN values are discarded). The correlation results concern classification performance and each of the 8 imbalance measures. Note that on the line ‘Correlation between f1 and f1’, the first ‘f1’ relates to the data complexity measure and the second to the classification performance (i.e., f1-score). This is obtained from step 7.

The protocol consists of three parts: i) benchmarking imbalance measures as indicators of classification difficulty on synthetic and real datasets (i.e., under controlled scenarios and uncontrolled ones), ii) investigating the relationship between imbalance measures and other data complexity measures, and iii) exploring the impact of each core component of SIMBA’s formula with an ablation study. Examples of the expected outcomes are presented in Figures 4 and 5. Note that if you have run the minimal examples and not the full-scale experiments, the resulting figures will not be identical. For instance, for the synthetic datasets, as they are run only 1 time and not smoothed over 100 runs, the Adj-IR and SIMBA will display a lot of variance due to the randomness of feature generation.

All the results from the correlation analyses are saved as.csv files in the results subfolder after steps 1 and 2 for synthetic datasets and after step 5 for real datasets. Similarly, the results of the data complexity analysis and the ablation study are saved as.csv files after steps 7 and 8, respectively. The resulting files from steps 1, 2, 5, and 8 are used for results visualization in steps 3, 4, 6, and 9, respectively. The expected resulting files are described in Table 1.

**Table 1:** Expected resulting.csv files for different steps of the protocol

Step	Resulting.csv file	Content
1	corr_synth_varying_data_*sID*_*X*.csv	Correlation coefficients between classification performance and imbalance measures over synthetic datasets with varying data distribution.
1	plot_synth_varying_data_*sID*_*X*.csv	Necessary information for the plots displayed in step 3.
2	corr_synth_varying_ft_*sID*_*X*.csv	Correlation coefficients between classification performance and imbalance measures over synthetic datasets with varying feature components.
2	plot_synth_varying_ft_*sID*_*X*.csv	Necessary information for the plots displayed in step 4.
5	real_*clf*_*X*.csv	For each real dataset: name, whether it is binary, score of each imbalance measure, and classification performance.
7	data_complexity_analysis_*clf*_*X*.csv	For each real dataset: name, score of each imbalance measure, classification performance, and score of each data complexity measure.
8	ablation_real_*clf*_*X*.csv	For each real dataset: name, score with each version of SIMBA, and classification performance.

*Naming conventions: sID* = scenario ID (‘a’, ‘b’, or ‘c’); *X*: evaluation metric (‘f1’ or ‘gmean’); *clf*: classifier (‘SVM’, ‘LDA’, ‘RF’, ‘kNN’, or ‘MLP’).

For the benchmark on synthetic datasets, steps 1 and 2 finish with the correlation results appearing in the terminal, as displayed in Figure 4A. Steps 3 and 4 show the trends followed by the imbalance measures on the different scenarios; each plot focuses on one imbalance measure. Figure 4B shows the trends of SIMBA for scenarios with varying data distribution, and Figure 4C for scenarios with varying feature components. To see the plots for all imbalance measures together, we refer to our related manuscript.^1^ For the benchmark on real datasets, the results are displayed per imbalance measure. Step 6 first presents the correlation results in the terminal (Figure 4E), then a figure appears with regression lines between the imbalance measure and the classification performance (Figure 4D). The figure is separated into three panels with all datasets, only binary ones, and only multiclass ones.

For the data complexity analysis (step 7), the results appear per data complexity measure in the terminal. For each data complexity measure, its correlation with classification performance and with 8 imbalance measures (namely, IR, Adj-IR, C1, C2, ID, LRID, IF, and SIMBA) is displayed, as shown in Figure 5C. To see the results for all data complexity measures, we refer to our related manuscript.^1^

For the ablation study, step 9 finishes with the correlation results appearing in the terminal, followed by a plot showing the regression lines for the 4 versions of SIMBA, as displayed in Figures 5A and 5B, respectively.

With these experiments, SIMBA shows to be a better indicator of classification difficulty in comparison to both other imbalance measures and data complexity measures. Indeed, with the correlation analysis on synthetic and real datasets, SIMBA follows coherent trends on synthetic data and consistently displays higher correlation with classification performance compared to other imbalance measures. Further, SIMBA appears to be independent of other data complexity measures with no strong correlations overall. Finally, the ablation study shows that both normalization and feature importance are key to quantify imbalance with a substantial correlation drop when any of these two core components are removed; however, feature redundancy does not appear crucial to the formula as it only slightly changes the resulting correlation coefficients. For more detailed results and conclusions, we refer to the related manuscript.^1^

## Limitations

The presented protocol consists of three parts: a benchmark on synthetic and real datasets (i), separately, a data complexity analysis (ii) and an ablation study for SIMBA’s formula (iii) on the real datasets. For the synthetic datasets, while the current scenarios vary in data distribution and feature components, they rely on certain fixed parameters (e.g., the number of classes in the datasets tested) and could be expanded to include a wider range of controlled variations. For the real datasets, pre-formatted collections from the UCI Machine Learning Repository^11^ and KEEL^12^ are used; future work could incorporate more complex datasets. The ablation study is constructed around SIMBA and its 3 core components. As such, to use it for a different formula, the ablation study must be adapted to the specific formula at hand.

The evaluation currently includes five classifiers and two classification performance metrics. This could be extended to include a broader set of algorithms and evaluation criteria, even though deep learning models were intentionally excluded because many of the 70 datasets contain limited sample sizes, a setting in which such models are prone to overfitting. Finally, the workflow was validated only on Windows OS. Although the dependencies are cross-platform, use on Linux or macOS has not been formally assessed and may require minor OS-specific adjustments.

## Troubleshooting

### Problem 1

When checking Python’s version is installed in this step, an error can be raised, or the wrong version of Python might appear.

### Potential solution

It is possible that Python was not properly installed, in which case you should try step 2 of the section ‘set up the coding environment’ again, and make sure to check the ‘Add Python to PATH’ option during installation, as displayed in Figure 1A. Close and reopen the terminal or reboot the computer before running step 3 of the section ‘set up the coding environment’ again. If multiple Python versions exist (no error raised, but the wrong version of Python appears in the terminal), try to modify the command line of step 3 of ‘Set up the coding environment’ with.

>python3.11 –-version

### Problem 2

**Figure 6:**
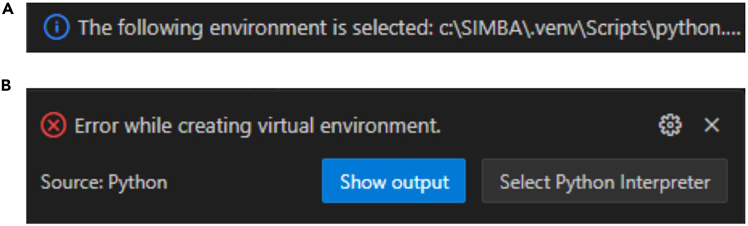
Outcomes of creating the virtual environment with dependencies (A) The virtual environment was successfully created with all the dependencies from requirements.txt. (B) One of the dependencies from requirements.txt could not be properly installed, hence there was an error in the creation of the virtual environment.

When creating the virtual environment here with the dependencies contained in *requirements.txt*, an error can be raised if one package cannot be properly installed. A pop-up window will appear on the bottom right of VS Code, as depicted in Figure 6.

### Potential solution

If that situation arises, go back to the creation of the virtual environment, but do not select *requirements.txt* as dependencies to install. Once the environment is created, activate it as described in step 5 of the ‘set up the coding environment’ section. For each line of the *requirements.txt* file, in the terminal, launch the command:

>pip install [insert package name]==[insert package version]

At least one of these commands will end up in an error message, corresponding to the package(s) that caused the problem in the first place. For this package, try to install it without specifying a version:

>pip install [insert package name]

Continue until all packages from the requirements.txt file are installed in the virtual environment.

### Problem 3

For each step, each time you launch:

>python ./main.py

an error might occur if any of the following are unrecognized: the specified action, the evaluation method (either f1 or gmean), the selected classifiers, the scenarios, or the number of repetitions for synthetic datasets. This issue can happen in steps 1.c., 2.c., 3, 4, 5.b., 6.b., 7.c., 8.c., and 9.

### Potential solution

**Figure 7:**
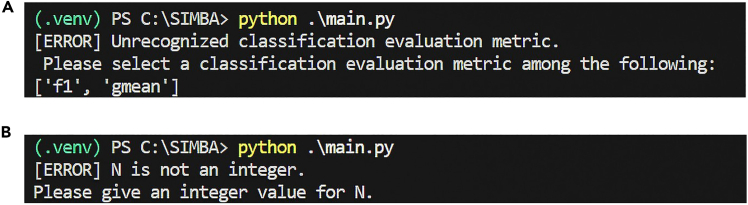
Examples of error messages when launching the main function (A) Error message when the specified evaluation method is unrecognized. (B) Error message when the number of repetitions (N) for synthetic datasets is assigned a value with an incorrect data type.

A message in the terminal starting with ‘[ERROR]’ will appear. If this happens, carefully read this message; it should clearly indicate which variable was not recognized (see Figure 7). Go back to the *main.py* file, to where this variable is declared, and change its value to fix the error.

### Problem 4

In the correlation analysis on synthetic datasets, an optional step mentions that other scenarios can be implemented. How to decide which new scenarios to implement?.

### Potential solution

• For scenarios where the data distribution varies, the number of classes and feature components must remain fixed. In this protocol, a total of 10 features are used, with 2 of them discriminative. To consider multiclass datasets, 3 classes are included in all scenarios. It is possible to choose a different number of classes; e.g., 2 for binary problems or more than 3 for potentially more complex classification tasks. When selecting the number of features, we recommend a sufficiently large set (e.g., at least 8 or 10) to prevent generating datasets that are overly simple to classify, regardless of data distribution.
• For scenarios where feature components vary, the data distribution remains fixed. In this protocol, as we considered multiclass datasets and examined imbalance measures, the datasets included 3 classes with 400 observations in class 1, 75 in class 2, and 100 in class 3. Both the number of classes and the number of observations per class can be modified. We recommend maintaining a sufficiently imbalanced data distribution to effectively observe the effects of varying feature components on the imbalance measures.

### Problem 5

In step 5 and step 6, when you launch:

>python ./main.py

with the action ‘real_data’ (or ‘real_data_plot’), an error might occur if one of the classifiers specified in the list of classifiers to use on line 101 (or line 110, respectively) from the *main.py* file is unrecognized.

### Potential solution

A message in the terminal starting with ‘[ERROR]’ will appear, similar to problem 3; it will clearly indicate that one of the classifiers was not recognized. Go to the list of classifiers in the *main.py* file, and check carefully the names of the classifiers that you have used, and change the incorrect names to fix the error.

### Problem 6

In the correlation analysis on real datasets, Data complexity analysis, and performing the ablation study sections, an optional step mentions that it is possible to add new datasets. Including additional real datasets can cause issues, namely because of the formatting. How to prevent errors when adding a dataset?

### Potential solution

It is important for the newly added dataset to respect the format of all other datasets. In particular, the dataset folder contained in the data folder should contain.

• One df_allfeatures.csv file for which each row is a data point and each column is a feature, except the first column, which corresponds to the id of the data point;
• One labels.csv file, for which each row is a data point. The first column corresponds to the id of the data point, and the second column is the label (encoded as an integer).

For any sample from the dataset containing missing values, delete this sample because some classifiers cannot handle them. Although this filtering may alter the class distribution, imbalance measures are computed after preprocessing, ensuring they reflect the data used in the classification task. For values that are nominal/categorical, encode them into integers. These two steps are necessary because five classifiers are used, among which some cannot handle missing values or non-numeric values.

To format the data, you can help yourself with the *data_processing.py* from the Zenodo repository.^10^

### Problem 7

In step 7, when you launch:

>python ./main.py

with the action ‘complexity’, in addition to the potential error shown in problem 3, another error might occur if the classifier or evaluation method specified for the function on line 124 is different from the one specified for the function on line 125 in the *main.py* file.

### Potential solution

**Figure 8:**
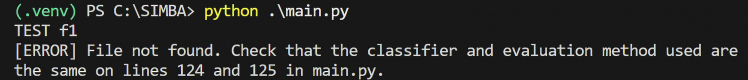
Example of an error message when running the data complexity analysis

A message in the terminal starting with ‘[ERROR]’ will appear, similar to problem 2; it will clearly indicate the variables that should be checked (Figure 8). Go to the indicated lines in the *main.py* file, and check carefully that the classifier and evaluation method used in both lines are identical. If not, change it appropriately to fix the error.

### Problem 8

If you run the action ‘ablation_plot’ (step 9) without having run the action ‘ablation’ (step 8) first, with the same specified classifier and evaluation method, a FileNotFound error will be raised.

### Potential solution

The error comes from the fact that the action ‘ablation_plot’ aims at visualizing the results obtained from the ‘ablation’ action. Once the ‘ablation’ action finishes running, a file of results is saved in the results subfolder. Its name contains the classifier and evaluation method used (e.g., *ablation_real_SVM_f1.csv*). Hence, if this error happens, check the presence of the file in the results folder, and the classifier and evaluation method that were used. Either run the ‘ablation’ action again (if the file you want is not in the results folder) or change the selected classifier and evaluation method to match the name of the results file.

## Resource availability

### Lead contact

Requests for further information, resources, and software should be directed to and will be answered by the lead contact, Julie R. Pivin-Bachler (julie@pivin-bachler.eu).

### Technical contact

Questions about the technical specifics of performing the protocol should be directed to and will be answered by the technical contact, Julie R. Pivin-Bachler (julie@pivin-bachler.eu).

### Materials availability

This study did not generate new unique reagents.

### Data and code availability

This paper analyzes existing, publicly available data from the UCI Machine Learning Repository^11^ and the KEEL Dataset Repository,^12^ as listed in the key resources table. The formatted datasets and source code are available in an open access deposition at Zenodo.^10^

## Acknowledgments

The authors thank the 10.13039/100032719Honda Research Institute in Japan for funding this research.

## Author contributions

Conceptualization, J.R.P.-B. and E.L.v.d.B.; methodology, J.R.P.-B. and E.L.v.d.B.; coding, J.R.P.-B.; writing – original draft, J.R.P.-B.; writing – review and editing, J.R.P.-B. and E.L.v.d.B.; supervision, E.L.v.d.B.; funding acquisition, E.L.v.d.B.

## Declaration of interests

The authors declare no competing interests.
